# The Application of rs-fMRI in Vascular Cognitive Impairment

**DOI:** 10.3389/fneur.2020.00951

**Published:** 2020-09-11

**Authors:** Ran Wang, Nian Liu, Yun-Yun Tao, Xue-Qin Gong, Jing Zheng, Cui Yang, Lin Yang, Xiao-Ming Zhang

**Affiliations:** Sichuan Key Laboratory of Medical Imaging, Medical Research Center, Department of Radiology, Affiliated Hospital of North Sichuan Medical College, Nanchong, China

**Keywords:** vascular cognitive impairment, resting-state functional magnetic resonance imaging, amplitude of low frequency fluctuation, regional homogeneity, functional network, default mode network, traumatic brain injury

## Abstract

The incidence of vascular cognitive impairment (VCI) has been increasing for years and has become a major disabling factor in middle-aged and elderly populations. The pathogenesis of VCI is unclear, and there are no standard diagnostic criteria. Resting-state functional magnetic resonance imaging (rs-fMRI) can be used to detect spontaneous brain functional activity in a resting state, which facilitates in-depth investigation of the pathogenesis of VCI and provides an objective reference for early diagnosis, differential diagnosis, and prognostic evaluation. This article mainly reviews the principle and analysis of rs-fMRI data, as well as the progress of its application for VCI diagnosis.

## Background

Vascular cognitive impairment (VCI) is a broad concept that refers to all forms of cognitive impairment (CI) associated with cerebrovascular diseases, which covers the full spectrum from vascular mild cognitive impairment (vascular MCI) to vascular dementia (VaD) and includes cases with mixed pathologies, such as mixed vascular and AD-type pathologies (1–3). According to the Vascular Impairment of Cognition Classification Consensus Study (VICCCS), VCI includes two types (4, 5): mild VCI and major VCI (VaD); major VCI includes the following four subtypes: post-stroke dementia (PSD), subcortical ischemic vascular dementia (SIVD), multi-infarct (cortical) dementia (MID), and mixed dementias (MixD). With the aging population, the incidence of dementia has increased over the years. VCI is the second most common cause of dementia, only after Alzheimer's disease (AD) (6, 7). VCI not only affects patients' quality of life but also causes a heavy burden to families and society.

Currently, the pathogenesis of VCI is not completely clear, and there is no unified diagnostic standard. The sensitivity and specificity of VCI diagnosis based on clinical manifestations and various scales are not high. The heterogeneous nature of cerebrovascular disease makes it challenging to clarify the pathological substrates of VCI. Studies indicated that the pathological substrates of VCI mainly involved arteriolosclerosis, small or lacunar infarcts, microinfarcts, perivascular space dilation, myelin loss, leptomeningeal cerebral amyloid angiopathy, and so on. In recent years, cerebral small vessel disease (SVD) has been recognized as an important substrate of CI. SVD is characterized by arteriolosclerosis, lacunar infarcts, cortical and subcortical microinfarcts, and diffuse white matter changes (including myelin loss and axonal abnormalities) (8–14). In addition, some biomarkers such as the cerebrospinal fluid (CSF) matrix metalloproteinases (MMPs), CSF/serum albumin quotient (QA), and possibly blood inflammatory cytokines and adhesion molecules, etc., can indirectly reflect the pathophysiological process of VCI (15–17).

MRI should be the first choice for patients with suspected VCI. The evaluation contents include brain atrophy, infarction, white matter hypertensities (WMHs), and hemorrhage (18). In recent years, fMRI development has provided important new research methods for studying the relationship between cerebrovascular diseases and CI. Resting-state functional magnetic resonance imaging (rs-fMRI) can be used to detect spontaneous brain functional activity in a resting state, which facilitates in-depth investigation of the pathogenesis of VCI and provides an objective reference for early diagnosis, differential diagnosis, and prognostic evaluation. This article mainly reviews the principle and analysis of rs-fMRI data, as well as the progress of its application for VCI diagnosis.

## Fundamental Principles of rs-fMRI

Blood-oxygen-level-dependent fMRI (BOLD-fMRI) can be used to acquire brain activity images based on hemodynamic changes in different functional brain areas (19). When the brain blood flow in the activation zone significantly increases, the blood oxygen level and relative proportion of oxygenated hemoglobin and deoxyhemoglobin will increase, resulting in changes in compliance in the corresponding region. Compliance changes can cause MRI signal variations. Brain maps with different functional states can be drawn through relevant data processing (20–23). BOLD-fMRI has mainly two modes: task-state and resting-state. Task-state fMRI requires task design for the subjects, and the subjects need to fully cooperate to accurately complete the task. rs-fMRI does not require specific task design for the subjects, and subjects only need to be awake with closed eyes, breathe calmly, minimize active and passive physical movements, and avoid any thinking activities. rs-fMRI detects spontaneous neuronal activity in the baseline state of the brain by MRI scan and determines the network connection of relevant brain regions, reflecting the spontaneous functional activities in the resting state (24). Compared with task-state fMRI, rs-fMRI has the characteristics of being relatively simple, easy to operate, easy to accept by subjects, and easy to conduct for large sample size studies. Because fMRI is non-invasive, has good temporal and spatial resolution, and can visibly reflect the features of relevant brain functional changes, it has been widely used in brain functional imaging studies.

## rs-fMRI Data Analysis

rs-fMRI mainly analyzes the abnormal activities of the brain region of the subject from three aspects: local brain function, functional connectivity (FC), and functional network. For local brain activity, the amplitude of low frequency fluctuation (ALFF) and regional homogeneity (ReHo) are used to analyze spontaneous neural activity in the local brain region from the perspective of functional separation. Brain FC analysis generally uses analytical methods that target all limited numbers of brain regions, including seed-based correlation analysis for the region of interest (ROI), independent component analysis (ICA), principal component analysis (PCA), dynamic causal analysis (DCM), and Granger causal analysis, among which seed-based correlation analysis and ICA are more commonly used. A brain functional network is a collection of functional dependencies among anatomically identified brain regions. It shows many topological properties, for example, small-world network attributes, among others. Recently, graph theory-based network analyses have been widely used in brain functional network research.

## ALFF

ALFF was proposed by Zang et al. (25) to reflect the intensity of spontaneous synchronized neural activity of various voxels in the low-frequency range (0.01–0.08 Hz) from the perspective of energy metabolism (25). Increased ALFF indicates an increase in excitability in the brain region. ALFF is correlated with cognitive function in the subject (26). ALFF is more sensitive to physiological noise. The fractional ALFF (fALFF), which is the ratio of the low-frequency power spectrum to the power spectrum of the entire frequency range, can significantly inhibit the physiological noise in the cistern in rs-fMRI and improve the sensitivity and specificity of spontaneous brain activity detection (27).

## ReHo

The ReHo method uses Kendall's coefficient concordance (KCC) to measure the consistency of time series between voxels in the brain. The ReHo map of the subject was obtained by calculating the KCC value of each voxel in the whole brain (28). ReHo reflects the functional status of the whole brain, but it cannot specifically indicate the activity status of each brain region. The increase in ReHo value means that the neuron activity tends to be synchronized in time, and vice versa, indicating that the neuron activity consistency is reduced, suggesting that neuron activity in the corresponding brain regions is abnormal.

## ICA

ICA is a type of data-driven method that has been widely used to analyze FC in the brain (29, 30). This method decomposes the signal into multiple spatially independent components and identifies a FC between brain regions with a relatively large signal projection on the same component. ICA is not necessary to preselect the seed-based region of interest and can separate the independent components corresponding to each functional network, as well as the impact of noise such as head motion and respiration on the signal. ICA can determine the spatial distribution of the network, but it cannot measure the connection strength of the brain regions.

## Seed-Based Correlation Analysis

Seed-based correlation analysis is the most basic method for early studies of FC, and it is also a common analytical method for rs-fMRI study of FC, which is a supplement to ICA. It has been adopted in many studies (31, 32). The seed-based correlation analysis first determines the seed-based coordinates of a network, extracts the BOLD signal from the seed area, and determines the temporal correlation between the ROI and other brain voxels.

This method can reflect the strength of association between brain regions and has the characteristics of being simple, sensitive, and easy to compare differences between groups. However, the analysis results depend on the selection of seed areas and cannot simultaneously process multiple systems (33).

## Graph Theory-Based Network Analyses

The brain is a complex and highly efficient network composed of spatially distributed but functionally connected brain regions. The brain network enables the coordination between neuron activities and between different brain regions. A complex network consists of nodes (representing the structural regions of the brain) and the edges connecting the nodes (34). The brain network has efficient small-world network attributes (35–41). The small-world network has a high clustering coefficient (Cp) and a short characteristic path length (Lp), allowing the transmission and processing of functional network information to be performed efficiently (35, 36). Small-world networks reflect brain functional differentiation and information integration, as well as the adaptability of the human brain to a variety of strong stimuli (37, 38). Threshold selection directly affects the statistical characteristics and topology of brain networks (39, 40). There are some groups of nodes in the network with tight internal connections but sparse external connections, called modules. The modularity Q reflects the level of functional separation of the brain network, and the modular structure allows a more detailed differentiation of the roles and statuses of different nodes. Graph theory analysis is a high-level network analytic method that is widely used in complex brain network research.

## Application of rs-fMRI in the VCI Study

### Local Brain Activity

Local brain activity abnormalities are associated with CI. In a resting state, the default mode network (DMN) is very active, and the activities of these brain regions significantly weaken under certain cognitive tasks (42). These regions include the medial prefrontal cortex (mPFC), posterior cingulate cortex (PCC), precuneus, anterior cingulate cortex (ACC), parietal cortex, and, in a minority of studies, also the hippocampus (43). Among the anatomical parcellation units, the ordering of the low-frequency oscillation (LFO) amplitudes is significant, showing a significant spatial distribution (44). DMN is closely related to the extraction of episodic memory, environmental alertness, cognition, and emotional processing. Many studies indicated that LFO amplitude measurement can describe the intergroup features of the rs-fMRI dataset and contribute to investigations of the pathogenesis of VCI (45–48). Liu et al. (45) investigated the ALFF alteration of whole brain in 30 patients with SIVD and 35 control subjects using structural MRI and rs-fMRI scan. Their study showed that the ALFF levels in the bilateral ACC, PCC, mPFC, inferior parietal lobe (IPL), occipital lobe, and adjacent precuneus were higher than the global mean ALFF levels in both groups. Compared to the controls, SIVD patients presented lower ALFF levels in the bilateral precuneus and higher ALFF levels in the bilateral ACC, left insula, and hippocampus. The ALFF values of the left insula were negatively correlated with the Montreal Cognitive Assessment (MoCA) and Mini-Mental State Examination (MMSE) scores of SIVD patients.

Yi et al. (46) investigated changes in functional amplitude of spontaneous LFO and FC density in 26 patients with subcortical vascular mild cognitive impairment (svMCI) and 28 healthy controls (HCs). They found that the svMCI-related changes were mainly located in the DMN. Compared with the HCs, the patients with svMCI presented decreased LFO amplitudes in the anterior part of the DMN and increased LFO amplitudes in the posterior part of the DMN.

**Figure 1:**
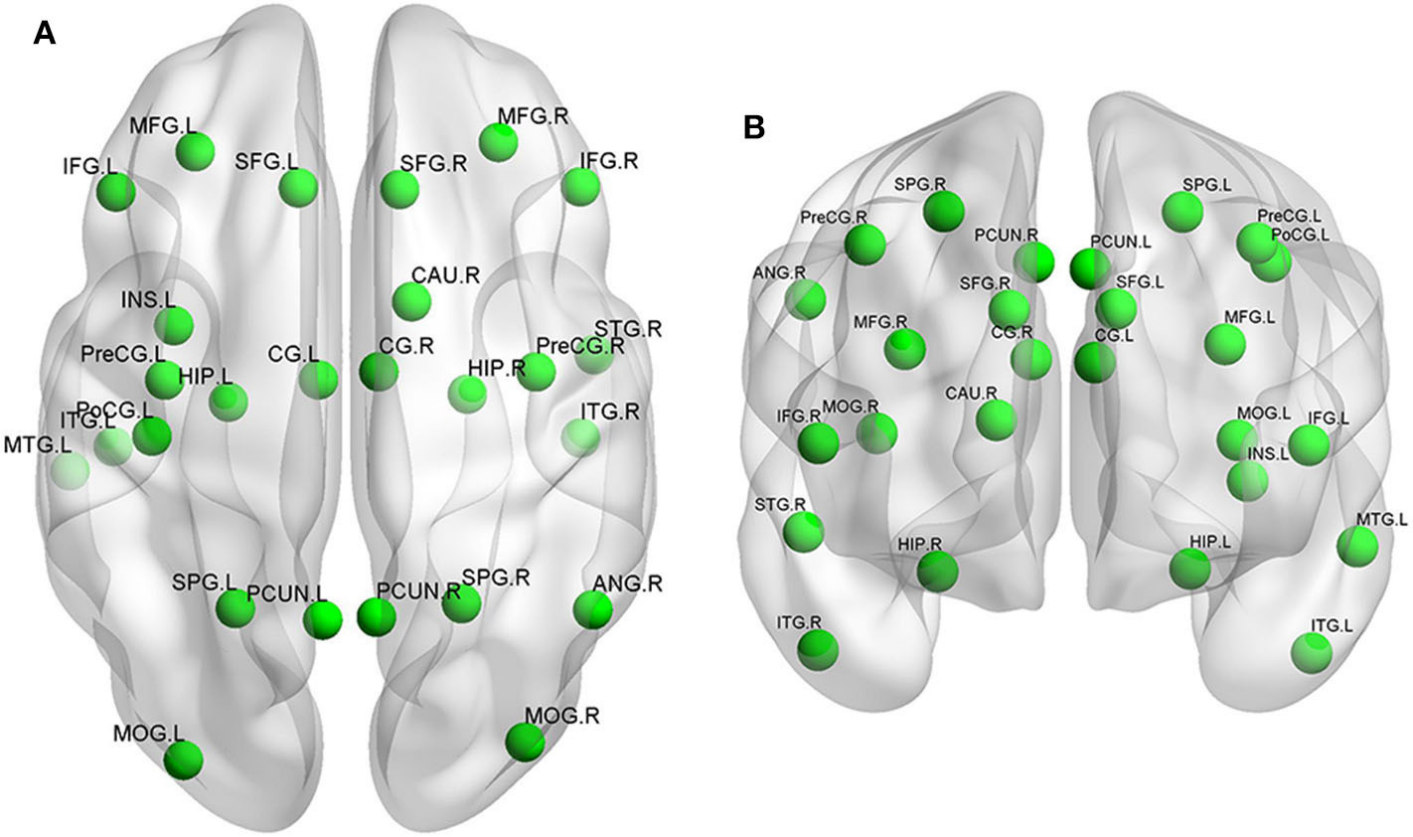
Neural networks involved in VCI mainly include the prefrontal cortex, temporal cortex, and hippocampus. The functional changes are shown in the **(A)** superior view and **(B)** anterior view. L, left; R, right; SFG, superior frontal gyrus; MFG, middle frontal gyrus; IFG, inferior frontal gyrus; PreCG, precentral gyrus; PoCG, postcentral gyrus; SPG, superior parietal gyrus; ANG, angular gyrus; PCUN, precuneus; MOG, middle occipital gyrus; STG, superior temporal gyrus; MTG, middle temporal gyrus; ITG, inferior temporal gyrus; INS, insular cortex; CG, cingulate gyrus; CAU, caudate; HIP, hippocampus.

ReHo values were highly correlated with cognitive function. Studies have shown that ReHo of related brain regions of VCI patient was significantly decreased (Figure 1). Peng et al. used ReHo analysis for post-stroke patients with poor cognitive function (PSPC) (49). Compared with the control group, PSPC patients had significantly reduced ReHo values in the bilateral anterior cingulate gyrus and left posterior cingulate gyrus/precuneus. Another study (50) explored the relationship of white matter lesions (WMLs) with CIs from the aspect of cortical functional activity in 16 patients with ischemic WMLs and 13 controls using a ReHo approach. This study showed regions with altered ReHo values in patients with ischemic WMLs to be involved in DMN, frontal–parietal control network (FPCN), dorsal attention network (DAN), motor network, and right temporal cortex. Moreover, some altered regions belonging to DMN, FPCN, and motor network were correlated with MMSE and MoCA scores significantly. It is very interesting to find that the decreased ReHo was mainly in the anterior brain regions while increased ReHo was in the posterior brain regions. This result may indicate a failure in downregulation of spontaneous activity in posterior regions. Similar results were obtained in the other studies (51, 52). The above results indicate that changes in ReHo values in related brain regions can be used as imaging markers for VCI.

### Brain FC

Because DMN FC abnormalities are closely related to CI, and PCC is the core part of DMN, PCC is often selected as the ROI to study its connection with other brain regions (53–55).

Carotid artery stenosis (CAS) without transient ischemic attack (TIA) or stroke has been previously considered asymptomatic. However, many studies have shown that asymptomatic CAS (aCAS) is not truly asymptomatic, and these patients are considered cognitively impaired in functional performance, psychomotor speed, and memory testing. Wang et al. (56) performed FC analysis in 19 aCAS patients and 24 HCs. Compared with the controls, aCAS patients exhibited significantly poorer performance on global cognition, memory, and executive function, and suffered decreased connectivity to the PCC in the anterior part of DMN. Sun et al. (57) investigated the changes in the resting state of 16 patients with VCI. The patients presented FC decrease in the left middle temporal gyrus, the left anterior cingulate/left middle frontal gyrus, the right caudate, the right middle frontal gyrus, and the left medial frontal gyrus/paracentral lobule. However, there were also some regions that showed increased FC, including the right inferior temporal gyrus, the left middle temporal gyrus, the left precentral gyrus, and the left superior parietal lobule.

Many studies have shown (58–61) that FC abnormalities in VCI subjects are closely related to CI (Figure 1). DMN is the key brain region for post-stroke CI. Yi et al. (46) investigated changes in FC density in patients with svMCI. They found that the FC of DMN continued to decline in terms of FC density. Ding et al. (59) investigated the differences of FC in the DMN in 18 stroke patients with and without post-stroke cognitive impairment (PSCI vs. Non-PSCI). They found that both PSCI and Non-PSCI patients showed significantly decreased FC in the PCC/PCu and increased FC in the mPFC as well as left hippocampus. However, Non-PSCI patients showed more significantly increased FC in the mPFC and hippocampus than PSCI patients did.

However, some studies (61, 62) have shown that VCI patients not only present an abnormal FC of the DMN but also show abnormal FC of other multiple resting state networks (RSNs). Li et al. (61) studied 21 TIA patients who suffered from an ischemic event and 21 HCs using cognitive tests, psychiatric tests, and fMRI. Their results showed that TIA patients showed both decreased and increased FC in DMN and self-referential network (SRN) and decreased FC in DAN, central-executive network (CEN), core network (CN), somato-motor network (SMN), visual network (VN), and auditory network (AN) than HCs did. This study indicated that TIA is a disease with widely abnormal brain networks. Similar results were obtained by Wang et al. (57). DMN is a collection of regions affecting cognitive recovery. Park et al. (63) for the first time examined longitudinal changes in the DMN during the 6 months after stroke. In their study, the stroke patients demonstrated obviously decreased DMN connectivity of the PCC, precuneus, medial frontal gyrus, and IPLs at 1 month after stroke, and the DMN connectivity of these brain areas was almost restored at 3 months after stroke, suggesting that the period is important for neural reorganization. The DMN connectivity of the dorsolateral prefrontal cortex in the contralateral hemisphere is significantly associated with cognitive function recovery, which may be a compensatory process to overcome the CI caused by brain injury (63). These findings will help to further understand underlying VCI mechanisms and suggest that resting-state network connectivity can serve as an imaging biomarker for VCI.

### Brain Functional Networks

Studies of cerebral leukoencephalopathy, stroke, and carotid stenosis with or without VaD leading to VCI disease (64–66) have shown that the decline in cognitive function was correlated to the abnormal small-world network attributes and topological parameters of the brain. Yu et al. (67) conducted graph theory-based network analyses of 23 subcortical VCI (SVCI) patients and 20 HCs. The results showed that the brain functional networks of the SVCI patients and control groups showed small-world attributes within the threshold range (0.15 ≤ sparsity ≤ 0.40). The global topological organization of the functional brain networks in SVCI was significantly disrupted. The reduction of the SVCI active area occurred mainly in the frontal lobe, while the subcortical area showed an increase in characteristic Lp, potentially compensating for the inefficiency of the functional network. Yi et al. (34) obtained similar conclusions.

## Application of rs-fMRI in Mild Traumatic Brain Injury (mTBI) and Other Cognitive Disorders

**Figure 2:**
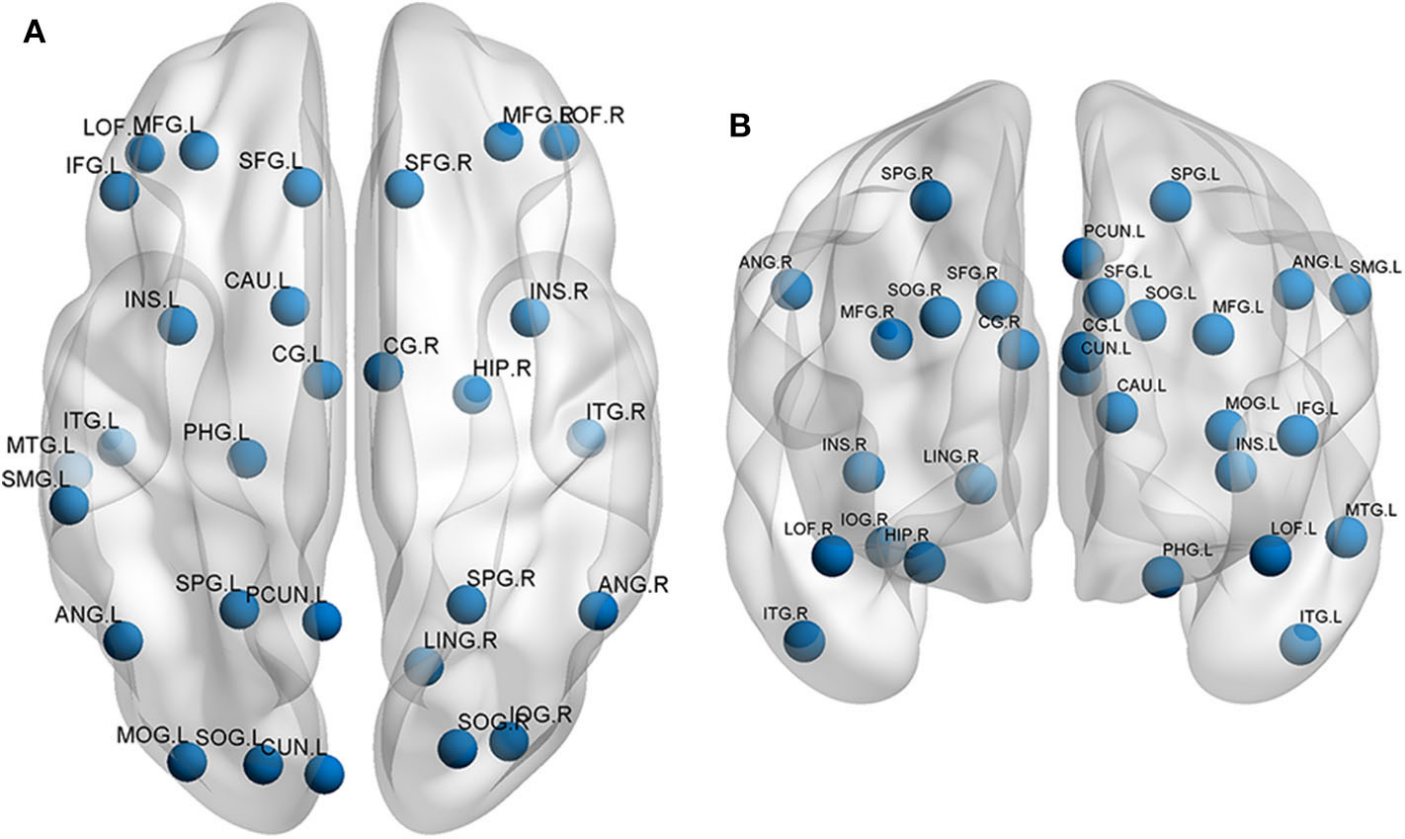
Neural networks involved in traumatic brain injury mainly include the prefrontal cortex, temporal cortex, occipital cortex, and cingulate gyrus. The functional changes are shown in the **(A)** superior view and **(B)** anterior view. L, left; R, right; SFG, superior frontal gyrus; MFG, middle frontal gyrus; IFG, inferior frontal gyrus; LOF, lateral orbitofrontal gyrus; SPG, superior parietal gyrus; SMG, supramarginal gyrus; ANG, angular gyrus; PCUN, precuneus; SOG, superior occipital gyrus; MOG, middle occipital gyrus; IOG, inferior occipital gyrus; CUN, cuneus; MTG, middle temporal gyrus; ITG, inferior temporal gyrus; PHG, parahippocampal gyrus; LING, lingual gyrus; INS, insular cortex; CG, cingulate gyrus; CAU, caudate; HIP, hippocampus.

In recent years, the incidence of TBI has increased. Although the majority of patients with mTBI had mild or no significant brain damage and the conventional imaging results were usually negative, some patients had a certain extent of CI. Neural networks involved in TBI mainly include the prefrontal cortex, temporal cortex, occipital cortex, and cingulate gyrus (68, 69) (Figure 2). One study (63) showed that patients with mTBI had a significant decrease in ALFF in the cingulate gyrus, middle frontal gyrus, and superior frontal gyrus; the cingulate gyrus ALFF was significantly positively correlated with the working memory index. In addition, FC of the thalamus, caudate nucleus, and right hippocampus of these patients was significantly reduced, and a significantly positive correlation between FC in the left thalamus and the left middle frontal gyrus and WMI. Zhu et al. (69) used rs-fMRI to study the longitudinal changes in function and structural connections of the DMN in mTBI patients at 24 h, 7 days, and 30 days. This study showed that the general trend of increased DMN FC occurred on the first day, which decreased significantly on day 7 and partially recovered on day 30.

In addition, studies have found that Parkinson's disease (PD), Huntington's disease, schizophrenia, major depressive disorder, type 2 diabetes mellitus (T2DM), chronic obstructive pulmonary disease (COPD), lung cancer following chemotherapy, hepatic encephalopathy, kidney deficiency syndrome, and other diseases can also cause CI (70–79), but the relationships between these diseases and cognitive function and the pathogenesis of CI are still unknown, necessitating further in-depth studies. rs-fMRI will play an important role in the exploration of these unknown areas.

## Conclusion

Brain dysfunction changes in VCI patients mainly occur in brain regions associated with DMN. FC changes are significant between different brain regions, and the global topological organization is extensively damaged. However, current rs-fMRI studies on VCI are in their infancy, and relatively few studies have been reported. There are many analytical methods for rs-fMRI data, and data processing procedures are complex. Current studies have not only utilized inconsistent research methods but also inconsistent imaging parameters. In future studies, rs-fMRI analysis should be standardized. In addition, rs-fMRI combined with other fMRI techniques and neuropsychological assessments can provide more complete data information. With the advancement of technology and in-depth studies, functional imaging markers will play a more important role in the pathogenesis, early diagnosis and differential diagnosis, early intervention, and prognostic assessment in VCI and post-mTBI CI.

## Author Contributions

RW, NL, Y-YT, X-QG, JZ, and CY contributed to the literature search and manuscript preparation. RW, NL, and LY revised the manuscript. X-MZ designed the research. All authors read and approved the final manuscript.

## Conflict of Interest

The authors declare that the research was conducted in the absence of any commercial or financial relationships that could be construed as a potential conflict of interest.
